# An Atypical Case of Taravana Syndrome in a Breath-Hold Underwater Fishing Champion: A Case Report

**DOI:** 10.1155/2013/939704

**Published:** 2013-07-22

**Authors:** Andrea Cortegiani, Grazia Foresta, Giustino Strano, Maria Teresa Strano, Francesca Montalto, Domenico Garbo, Santi Maurizio Raineri

**Affiliations:** ^1^Department of Biopathology and Medical and Forensic Biotechnologies (DIBIMEF), Section of Anaesthesiology, Analgesia, Emergency and Intensive Care, Policlinico “P. Giaccone,” University of Palermo, via del Vespro 129, 90127 Palermo, Italy; ^2^Hyperbaric Therapy Unit, ARNAS Civico, Piazzale Liotti, 4, 90127 Palermo, Italy

## Abstract

Dysbaric accidents are usually referred to compressed air-supplied diving. Nonetheless, some cases of decompression illness are known to have occurred among breath-hold (BH) divers also, and they are reported in the medical literature. A male BH diver (57 years old), underwater fishing champion, presented neurological disorders as dizziness, sensory numbness, blurred vision, and left frontoparietal pain after many dives to a 30–35 meters sea water depth with short surface intervals. Symptoms spontaneously regressed and the patient came back home. The following morning, pain and neurological impairment occurred again and the diver went by himself to the hospital where he had a generalized tonic-clonic seizure and lost consciousness. A magnetic resonance imaging of the brain disclofsed a cortical T1-weighted hypointense area in the temporal region corresponding to infarction with partial hemorrhage. An early hyperbaric oxygen therapy led to prompt resolution of neurological findings. All clinical and imaging characteristics were referable to the Taravana diving syndrome, induced by repetitive prolonged deep BH dives. The reappearance of neurological signs after an uncommon 21-hour symptom-free interval may suggest an atypical case of Taravana syndrome.

## 1. Introduction

Dysbaric accidents (DA) are usually referred to continuous air-supplied dives. Nonetheless diving accidents are known to occur also among breath-hold (BH) divers [1–6]. BH diving accidents include Taravana syndrome (TS), firstly described by Cross in 1965 [1]. He reported professional BH pearl divers in the Tuamoto Archipelago, in the South Pacific, presenting neurological disorders such as dizziness, vertigo, crossed sensory numbness, nausea, euphoria, dysarthria, hemiparesis, unconsciousness, and even sudden death, after repetitive BH dives with short surface intervals. Since Cross' description, several DA have been reported after repeated BH dives. In some cases the symptoms were sudden, occurring as the divers left the water, whereas in other cases they appeared 1-2 h later, depending on the dive profile [6].

We report an atypical case of TS occurred in an underwater fishing champion, previously in a good health, referred to the Hyperbaric Oxygen Therapy (HBOT) Unit of a general teaching hospital in Palermo, Italy. In this case, the appearance of neurological symptoms was followed by generalized tonic-clonic seizure and coma after an unusual 21-hour interval from the dives.

## 2. Case Report

A 57-year-old man, underwater fishing champion, non smoker and previously in a good health, started a dive session in a well-known dive site in the Mediterranean sea (Capo Gallo, Palermo, Italy) at 10:30 until 13:00 on a summer September day. He was correctly weighed and underwater conditions were optimal for diving (water temperature at 25°C). He performed 19 dives to a mean seawater depth of 30–35 meters sea water (msw) over 150 min. Each time in depth lasted 2 min and 10 s to 2 min and 50 s; the surface interval between dives was 1 min to 1 min and 30 s. During the 16th dive, a left frontoparietal pain occurred and, in the subsequent dives, he complained dizziness, blurred vision, and sensory numbness. After interrupting the section and reaching the surface, he called the HBOT Unit doctor and described what had happened. The doctor suspected a case of TS on the basis of both diving session characteristics and neurological symptoms and suggested him to go to the hospital. Nonetheless, the diver decided to come back home because of symptoms' regression.

**Figure 1:**
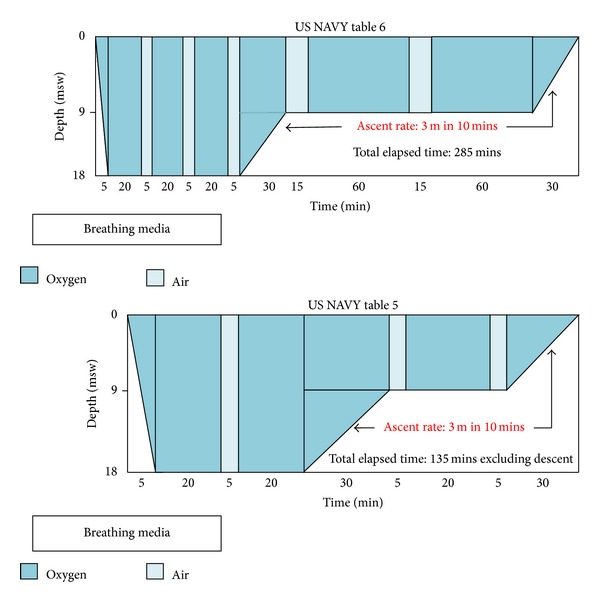
HBOT US NAVY table 6 and table 5. US NAVY table 6 consists of a compression phase about 5 minutes long to depth of 18 msw under 100% oxygen and 4 oxygen cycles lasting 20 minutes each with short air intervals. Then, the patient is decompressed to about 9 msw and exposed to 2 oxygen cycles lasting 60 minutes each and slowly returned to surface pressure. The total elapsed time is about 285 min (4 hrs 45 min). US NAVY table 5 is similar to table 6 but with shorter and lesser oxygen cycles; the total elapsed time is about 135 min, excluding descent. Adapted from http://www.londondivingchamber.co.uk/.

Next morning, at 6:00 he went for his usual 8 km run and the same symptoms of the day before reappeared. At 12:00, he arrived by himself to the Emergency Room. During the triage process, the staff assigned him a “yellow code” (meaning intermediate criticalness, not life-threatening conditions but requiring examination as soon as possible, frequent retriage, and hospital care). While waiting for being examined, he presented a generalized tonic-clonic seizure. He was promptly admitted to the Emergency and Critical Care Room where seizures ended in a few minutes without any specific pharmacological therapy. He was examined and his vital parameters were monitored (heart rate: 112 bpm, SpO_2_: 85% despite administration of 8 L/min of oxygen via Venturi mask, noninvasive blood pressure: 132/82 mmHg). The neurological assessment revealed a Glasgow Coma Scale value of 5 (eyes: 1; verbal: 1; motor: 3) and bilaterally isochoric, isocyclic, and reactive to light pupils. A blood-gas analysis revealed the following: pH: 7.33, PaCO_2_: 32 mmHg, PaO_2_: 72 mmHg, HCO_3_^−^: 20 mEq/L, lactate: 2.8 mmol/L, base excess: −2.2. An orotracheal intubation was performed and mechanical ventilation was started. A 12-lead ECG was obtained and did not present pathological findings. A complete blood count, hematocrit, hemostatic tests, and blood chemistry were within normal range. A brain computerized tomography (CT) scan without contrast agent was then performed showing two hypodensity areas in the left subcortical temporal-parietal region and in the right parietal region, both referable to recent ischemic lesions. A DA was suspected on the basis of clinical and imaging findings and the recent diving session reported by relatives and HBOT doctor. Thus, the patient was referred to the HBOT Unit to start a prompt treatment. HBOT was composed of one session using US NAVY table 6 at first, and two sessions of US NAVY table 5 within 12 and 36 hours (Figure 1). Patient's general conditions rapidly improved allowing weaning from mechanical ventilation and extubation during the last ascent of the first HBOT session.

**Figure 2:**
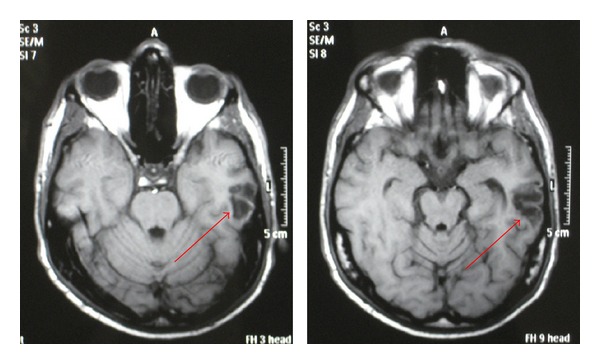
Brain MRI examination (axial T1-weighted sequence). The arrows point to an hypointensity area mainly involving left temporal subcortical white substance. It is likely to be an ischemic lesion with partial hemorrhage. Day 1 after diving session.

The patient was admitted to the Intensive Care Unit (ICU) and a thoracic CT scan and a brain magnetic resonance imaging (MRI) were obtained. The thoracic CT scan showed a ground-glass pattern particularly in the right lung. Brain T1-weighted MRI disclosed a subcortical hypointense area in temporal region corresponding to infarction with partial hemorrhage (Figure 2).

Echocolor Doppler of supra-aortic vessels was performed and did not reveal any abnormality. A transthoracic and a transesophageal echocardiographies (with contrast agent injection) were performed and no signs of patent foramen ovale were detected.

At 24 hours from the last HBOT session, the patient started the consolidation therapy, consisting of 10 HBOT sessions at 15 meters of sea water (msw), twice a day for 5 days. After 5 days from the admission, he was discharged from the ICU and admitted to the Neurology ward and then he left the hospital on day 10. A brain MRI was performed after hospital discharge showing a cortical hypointense area on T1-weighted and FLAIR images corresponding to loss of substance (3 cm × 2 cm) probably due to thromboembolic lesion in the left subcortical temporal area. Moreover, a hyperintense edge, due to glial phenomenon, and fair expansion of ventricular system, due to atrophic phenomena, appeared at FLAIR sequences. Actually, the patient has completely recovered from the neurological deficits and no apparent signs or symptoms remain. He restarted his diving activity as a great underwater fishing champion and no other accidents occurred.

## 3. Discussion

In this case, a Taravana syndrome was suspected because of the previous healthy status of the diver, the absence of known risk factors for stroke, clinical manifestations, diving session characteristics, and the prompt response to HBOT as well. Kohshi et al. [4, 5] described stroke-like events in Japanese ama and BH diving causing neurological impairment, which were also reported by several authors. Magno et al. [3] described 4 divers presenting neurological findings followed by total recovery. These disorders could be referable to the spectrum of decompression illness (DCI), covering both arterial gas embolism and insitu bubble formation, [7] and the mechanisms of brain damage are still uncertain [4, 8]. Many hypotheses have risen to explain the physiopathologic aspects linking BH to continuous air-supplied diving DA. During a series of BH dives, tissues reach a state of effective equilibrium between gain of nitrogen during time in depth and loss during surface intervals. The nitrogen tension will oscillate about a steady mean value. This value is equal to that which tissues reach in complete equilibration with air during a continuous air-supplied dive to a specific depth, called equivalent depth (ED), expressed as a percent of real depth. ED is determined by the ratio of surface intervals to time in depth. Considering that, for every ratio value, there is a specific ED. If the ratio is equal to 1, ED is 50% of real depth. For example, a series of BH dives at 33 msw with surface intervals both of 90-second long would be equal to a continuous dive to 16.5 msw depth. Thus, when BH divers perform repetitive and prolonged deep dives, nitrogen silent microbubbles could be released continuously from the venous side of tissues, according to Boyle-Mariotte law, and reach the cerebral arteries via uncertain circumstances, probably involving preexisting right-to-left shunt or barotrauma induced alveolar-capillary membrane injury, impairing the blood-brain barrier. Then, aggregation of microbubbles could form thrombi causing cerebral embolism [8–10]. In our patient, brain lesions demonstrated by MRI suggested a vascular pathogenesis with occlusion of cerebral arteries in terminal and border zone, probably due to nitrogen bubbles that accumulated in repeated and prolonged BH dives, without adequate recovery time on seawater surface. During the 16th dive, nitrogen bubbles were probably released abruptly from fatty tissues. The nitrogen, normally exchanged by breathing, gradually accumulated in blood vessels and flowed in larger bubbles that occluded arteries causing ischemic lesions in central nervous system [5, 11].

Our patient showed characteristic computerized tomography (CT) images as “ground-glass lung.” Pulmonary barotrauma of descent (also known as “lung squeeze”) occurs in BH divers when total lung capacity (TLC) decreases reaching the residual volume (RV). Thus, as the diver descends at a certain depth, gas in the lung is compressed and the TLC decreases as a consequence of increasing pressure [11]. At greater depths, the high negative transthoracic pressure, which develops as the diver passes 30 msw of depth, and the chest wall approaching its elastic limit both draw about 1 L of blood into the thorax [12]. Consequently, pulmonary capillaries bulge prominently into the alveolar spaces and replace air. It results in a decrease of RV and in extending depth limit [6]. Moreover, cold exposure increases preload and afterload by vasoconstriction, and exercise determines an increase in cardiac output, both involving a central blood pooling [12]. A combination of all these mechanisms, as occurs during diving, could be responsible for an excessive rise in pulmonary capillary pressure that can disrupt the blood-gas barrier and cause alveolar edema or hemorrhage and possible paradoxical gas embolism [10].

Several risk factors concerning diving session patterns have been proposed [6]. Cross [1] reported that pearl divers in Mongareva, a lagoon next to Tuamoto Archipelago, never developed TS. He observed that Mongareva's divers used the same diving techniques but with longer surface intervals. Since Cross' observation, others factors have been suspected and are shown in Table 1.

The diagnosis of DA is based on anamnesis and characteristic symptoms and signs. It is a mainly clinical diagnosis and there are no specific laboratoristic or radiologic tests, except for recompression that is both diagnostic and therapeutic. The ability to perform prerecompression diagnosis is usually limited by the need for urgent HBOT. In our case, the diagnosis of TS was strongly suspected on the basis of the absence of previous neurological signs and symptoms in anamnesis, the healthy state of the patient before the diving session, the absence of risk factors for stroke, and the characteristics of the diving session as well. The appearance of neurological signs and symptoms immediately after reaching seawater surface could be thought as another line of evidence. Myelopathy, skin rash, and joint pain may be associated with DCI but their absence could not exclude it [4, 13]. Echocolor Doppler, echocardiography, and transesophageal echocardiography showed the absence of lesions in the supraaortic vessels and of a patent foramen ovale. The apparent lack of right-to-left shunt may be consistent with the hypothesis of a paradoxical gas embolism because of alveolar-capillary barrier injury, as suspected on the basis of thoracic CT scan findings [4, 6].

Brain imaging may have a key role in the diagnosis of neurological involvement during DCI. Kohshi et al. [5] observed MRI images of multiple infarction in the terminal and border zone of cerebral arteries, where perfusion is poorest, in Japanese ama divers experiencing stroke-like symptoms. Although MRI is generally regarded as a sensitive method of detecting recent ischemic brain lesions, only one third of patients with transient ischemic attacks have lesions detectable by MRI [14]. Furthermore, MRI has a low sensitivity in the diagnosis of acute neurological DCI [15]. Vann et al. [7] reported MRI failure to find pathological remarks in a highly suspected TS case, probably consistent with the short duration of symptoms and the reversibility of MRI lesions associated with transient circulatory disturbances due to cerebral arterial embolism [8].

Even though a lot of elements support the diagnosis of TS, there are no lines of evidence in the literature about delayed symptoms in TS cases. Our patient presented the same neurological symptoms he had experienced after the diving session plus seizures and coma after 21 hours. Thus, it seems likely that both events had the same pathophysiologic cause. 

In conclusion, despite the lack of delayed symptoms cases of TS in the literature and considering the prompt and complete recovery of the patient, we suggest that a delayed occurrence of neurological impairment in a BH diver consistent with DA should be treated by HBOT as classic TS cases.

## Figures and Tables

**Table 1:** Several diving characteristics could be involved in the pathophysiology of DA during BH dives. Short surface intervals, high depth, and number and frequency of repeated dives appear to increase the risk of DA. Other elements as excessive hyperventilation before dives, intense physical exercise, and physiologic responses to the underwater environment could be considered as promoting factors.

BH diving characteristics related to DA development
Hyperventilation before dives
Rapid descent rate
Long time in depth
Short surface interval
Ratio of surface intervals to time in depth
High depth
Coldness
Intense physical exercise
Dehydration
